# The Central Role of HLA Class II-Restricted Helper Neoantigen Vaccines in Cancer Immunotherapy

**DOI:** 10.3390/cancers18152461

**Published:** 2026-07-31

**Authors:** Takafumi Morisaki, Takashi Morisaki

**Affiliations:** 1 Department of Cancer Immunotherapy, Fukuoka General Cancer Clinic, Fukuoka 812-0018, Japan; 2 Department of Surgery and Oncology, Graduate School of Medical Sciences, Kyushu University, Fukuoka 812-8582, Japan

**Keywords:** neoantigen, HLA class II, dendritic cell, vaccine

## Abstract

The discovery of neoantigens and their application in cancer vaccines have brought about a paradigm shift in cancer immunotherapy, similar to the way in which immune checkpoint inhibitors redefined therapeutic strategies for cancer treatment. Early neoantigen vaccine approaches primarily focused on eliciting HLA class I-restricted CD8^+^ cytotoxic T lymphocyte (CTL) responses, whereas it has become clear that such strategies alone may be insufficient to establish durable and effective antitumor immunity. Increasing attention has therefore shifted toward HLA class II-restricted neoantigens (“helper neoantigens”) that activate tumor-specific CD4^+^ helper T cells. Recent studies have revealed that neoantigen-reactive CD4^+^ helper T cells play a central role in coordinating antitumor immune responses through dendritic cell licensing, thereby sustaining CD8^+^ CTL function, preventing T-cell exhaustion, and promoting the generation of long-lived memory T cells. These findings have highlighted the pivotal role of helper neoantigens in shaping vaccine efficacy. In this review, we discuss the mechanistic basis underlying the contribution of helper neoantigens to tumor immunity and highlight recent advances in the real-world application of helper neoantigen-based vaccine strategies.

## Simple Summary

Neoantigens are tumor-specific molecules generated by cancer-associated mutations and are promising targets for personalized cancer vaccines. Early neoantigen vaccines mainly focused on activating CD8^+^ cytotoxic T cells; however, growing evidence indicates that effective and durable antitumor immunity also requires CD4^+^ helper T cells. HLA class II-restricted neoantigens (“helper neoantigens”) activate tumor-specific CD4^+^ helper T cells that support cytotoxic T-cell function, promote immune memory, and prevent T-cell exhaustion. Recent studies have identified helper neoantigens as key contributors to vaccine efficacy. In this review, we summarize the mechanisms by which helper neoantigens enhance antitumor immunity and discuss recent advances in helper neoantigen-based cancer vaccines. These findings may help guide the development of next-generation personalized cancer immunotherapies.

## 1. Introduction

The identification of tumor-specific neoantigens has led to a paradigm shift in cancer immunotherapy and paved the way for highly personalized therapeutic approaches [1,2]. Importantly, it has become clear that the activation of neoantigen-reactive T cells contributes substantially to the efficacy of immune checkpoint inhibitors (ICIs) [3,4,5,6]. Advances in next-generation sequencing technologies and artificial-intelligence-based computational epitope prediction have enabled the identification of patient-specific neoantigens and facilitated the development of highly personalized neoantigen vaccines capable of eliciting robust antitumor T-cell responses [7,8,9]. Based on the view that CD8^+^ cytotoxic T lymphocytes (CTLs) are the main mediators of antitumor immunity, early neoantigen vaccines focused predominantly on human leukocyte antigen (HLA) class I-restricted neoepitopes and the induction of tumor-specific CD8^+^ CTL responses [6,8,9]. Although these strategies generated neoantigen-specific CD8^+^ CTL responses, their clinical efficacy was limited.

Persistent antigen stimulation within the tumor microenvironment (TME), immune checkpoint signaling, and multiple immunosuppressive cellular mechanisms promote CD8^+^ CTL dysfunction and exhaustion, thereby preventing durable antitumor immunity [10,11,12]. In addition, tumor-intrinsic mechanisms, including antigen loss, defects in antigen processing and presentation pathways, and epigenetic changes, further undermine the effectiveness of therapeutic strategies that rely predominantly on CD8^+^ CTL responses [13,14]. These findings highlight the fundamental challenges of vaccine strategies that depend exclusively on HLA class I-restricted neoantigens.

Recent studies have changed our understanding of the cellular mechanisms underlying effective antitumor immunity by highlighting the central role of CD4^+^ helper T cells. Upon recognition of peptides presented by HLA class II molecules, CD4^+^ helper T cells orchestrate multiple aspects of adaptive immunity through diverse functions [15,16,17,18]. These include dendritic cell (DC) licensing through CD40–CD40L interactions, enhancement of CD8^+^ T cell priming and expansion, promotion of long-lived memory T cell differentiation, and production of essential cytokines such as interleukin-2 (IL-2) and interferon-γ (IFN-γ). In addition to these helper functions, CD4^+^ helper T cells modulate the composition and functional state of the TME by regulating myeloid cells and, under certain conditions, exerting direct cytotoxic activity against tumor cells.

Consistent with these observations, accumulating studies indicate that HLA class II-restricted neoepitopes (referred to hereafter as “helper neoantigens”) rather than HLA class I-restricted neoepitopes play a critical role in inducing therapeutic efficacy in neoantigen-based immunotherapy. Vaccines incorporating helper neoantigens elicit neoantigen-specific CD4^+^ helper T-cell responses and have demonstrated consistent superior efficacy compared with vaccines targeting HLA class I-restricted neoantigens alone. Mechanistically, helper neoantigen vaccines sustain CD8^+^ CTL functionality, limit T-cell exhaustion, and promote the generation of memory T cells, all of which promote efficient and durable antitumor immune responses [16,17]. Moreover, CD4^+^ helper T-cell-mediated immune regulation reshapes the TME into a more immunologically permissive state.

Collectively, these findings establish helper neoantigens as central players of effective antitumor immunity rather than merely supportive components of antitumor immune responses. By coordinating interactions among CD4^+^ helper T cells, DCs, and CD8^+^ CTLs, helper neoantigens promote sustained immune surveillance, durable immunological memory, and effective remodeling of the TME. The incorporation of helper neoantigens into cancer vaccine platforms therefore represents not simply an additional improvement but a paradigm shift in neoantigen vaccine design. In this review, we discuss the biological basis of helper neoantigen-mediated immunity, summarize recent advances in the development and clinical application of helper neoantigen vaccines, and outline emerging principles for the rational design of next-generation helper neoantigen-based vaccines.

## 2. The Discovery of Neoantigens and Immune Checkpoint Inhibitors

Initially, neoantigens were defined as tumor-specific mutant peptides derived from single nucleotide variants that give rise to single amino acid substitutions and are recognized by CD8^+^ T cells in an HLA class I-restricted manner.

One of the earliest reports of a tumor neoantigen vaccine was presented by Schreiber and colleagues in 1995 [19]. Using a murine sarcoma model, the authors identified a point mutation in the L9 protein that resulted in the substitution of histidine for leucine at position 47, generating a mutant peptide capable of eliciting a CD8^+^ CTL response and mediating tumor rejection. This study provided the first direct evidence that a tumor-specific mutation could give rise to an immunogenic antigen recognized by the adaptive immune system.

Subsequent studies identified additional mutation-derived antigens in experimental models, but the clinical relevance of neoantigens remained uncertain until the early 2000s. A major advance came in 2003, when Huang and colleagues reported the first evidence of neoantigen-specific T-cell responses in a patient with cancer [20]. In a melanoma patient who achieved complete regression following adoptive transfer of tumor-infiltrating lymphocytes, the investigators identified a mutated peptide derived from CDKN2A as the target of tumor-reactive CTLs. This finding demonstrated that naturally occurring T-cell responses against mutation-derived antigens could contribute to tumor eradication in humans. Nevertheless, the field at that time remained largely focused on shared tumor-associated antigens as vaccine targets, whereas patient-specific neoantigens were generally viewed as difficult to exploit therapeutically because of their individualized nature.

The significance of neoantigens was not fully appreciated until the advent of ICIs. Early clinical observations suggested that tumors with high mutational burdens were more likely to respond to ICIs; however, subsequent studies revealed that mutational burden itself was an imperfect surrogate for immunogenicity. From 2014 onwards, accumulating evidence indicates that the quantity and quality of neoantigens, as well as the magnitude of neoantigen-specific T-cell responses, are closely associated with therapeutic outcomes following exposure to ICIs [3].

McGranahan and colleagues analyzed patients with melanoma and non-small-cell lung cancer treated with ICIs and demonstrated that clinical responses were accompanied by the expansion of neoantigen-reactive T-cell populations [4]. Importantly, treatment efficacy correlated more strongly with the presence of clonally expressed immunogenic neoantigens and the expansion of corresponding T-cell populations than with the absolute number of predicted neoantigens alone. These findings highlighted the importance of neoantigen quality and immune recognition over neoantigen quantity.

Further evidence for the central role of neoantigens following treatment with ICIs was provided by Luksza and colleagues, who developed a mathematical framework to quantify neoantigen fitness [21]. This model integrated multiple parameters, including predicted HLA-binding affinity, the likelihood of T-cell receptor recognition, and the clonal distribution of neoantigen-expressing tumor cells. Across multiple patient cohorts, neoantigen fitness scores were strongly associated with clinical outcomes following ICI treatment, providing a mechanistic explanation for the relationship between neoantigen-directed immunity and therapeutic efficacy. Collectively, these studies established neoantigens not merely as biomarkers of tumor mutational burden but as functional determinants of antitumor immune responses and key mediators of successful cancer immunotherapy.

## 3. Limitations of Vaccines Using Cancer-Associated Antigens Recognized by CD8^+^ CTLs

Over the past two to three decades, multiple clinical trials have evaluated cancer vaccine strategies targeting tumor-associated antigens, including differentiation antigens, overexpressed self-antigens, and cancer-testis antigens presented by HLA class I molecules on tumor cells; however, these approaches have demonstrated only limited clinical efficacy [14,22,23]. Notably, although such vaccine strategies showed in vitro ability to induce HLA class I-restricted CD8^+^ T cell responses capable of recognizing and killing tumor cells, these immune responses have not consistently translated into meaningful clinical benefit. These findings suggest that CD8^+^ CTLs alone are insufficient to mediate durable and effective antitumor immunity in human cancers. Similar limitations have been observed in the context of neoantigen-based vaccines. Clinical trial data indicate that HLA class I-restricted neoantigen vaccines generally induce only modest tumor regression, and that meaningful therapeutic activity is often dependent on combination with ICIs [7]. To date, most neoantigen vaccine design strategies have relied primarily on in silico prediction algorithms optimized for HLA class I binding affinity. However, the accumulating evidence on the limited efficacy of CD8^+^ CTL-centric approaches underscores the need to reconsider current vaccine design paradigms. In particular, these observations highlight the necessity for alternative strategies that extend beyond HLA class I-restricted CD8^+^ CTL responses to achieve greater clinical benefit.

## 4. Dendritic Cell Licensing by CD4^+^ Helper T Cells

Unlike CD8^+^ CTLs, CD4^+^ helper T cells generally do not exert antitumor effects through direct recognition of tumor-associated antigens presented on tumor cells, except when tumor cells express HLA class II molecules. Consequently, CD4^+^ helper T cells were long thought to contribute to antitumor immunity primarily through indirect mechanisms, including the secretion of cytokines and other immunomodulatory factors. However, recent advances have fundamentally reshaped our understanding of the role of CD4^+^ helper T cells in antitumor immunity. Although CD4^+^ helper T cells were historically viewed as mediators of antitumor responses through indirect mechanisms, accumulating studies now indicate that their primary function lies in the orchestration of DC-dependent immune responses. In particular, the activation and licensing of DCs by CD4^+^ helper T cells have emerged as central processes governing the generation of effective antitumor immunity. The mechanisms by which DCs prime CD8^+^ CTLs through the presentation of tumor-associated antigens on HLA class I molecules are well established. However, effective CTL priming requires more than antigen recognition alone. During the priming of CD8^+^ T cells by HLA class I-restricted neoantigens, the absence of CD4^+^ helper T-cell signals accelerates the acquisition of dysfunctional or exhausted T-cell states [11,12]. This observation highlights the indispensable role of helper T cells in shaping the quality and durability of CD8^+^ CTL responses.

Mechanistically, DCs activate CD4^+^ helper T cells through the presentation of HLA class II-restricted peptides. In turn, activated helper T cells deliver licensing signals to DCs via CD40L–CD40 interactions, thereby enhancing their antigen-presenting and T-cell-priming capacities [15,16,17]. Licensed DCs subsequently promote the expansion and maintenance of CD8^+^ CTL responses through CD70–CD27-mediated co-stimulation. Beyond sustaining CTL activation, CD4^+^ T cell-help and licensed DCs also program multiple aspects of CD8^+^ CTL functionality, including the induction of chemokine receptors such as CXCR4 and CX3CR1, thereby enhancing their migratory capacity and facilitating infiltration into tumor tissues, the upregulation of cytotoxic effector molecules, such as granzyme B and IFN-g, and the suppression of inhibitory receptors such as PD-1, BTLA, and LAG-3 [16,24]. Such improvement of the tumor immune microenvironment is also expected to suppress the induction of regulatory T cells (Tregs).

**Figure 1:**
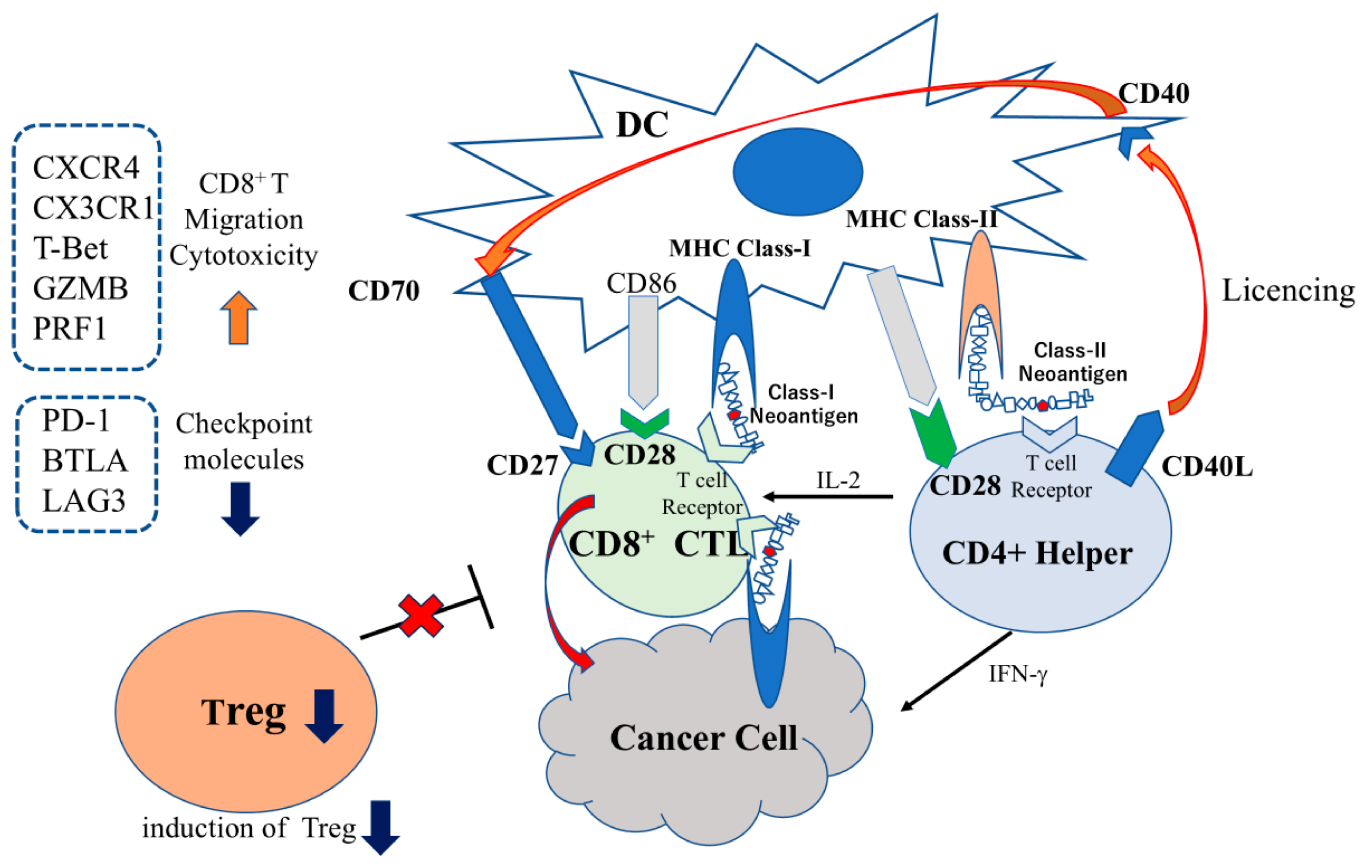
Neoantigen-reactive CD4^+^ helper T cell-mediated licensing of dendritic cells for CD8^+^ T cell activation. CD4^+^ helper T cells recognizing HLA class II neoantigens presented by mature dendritic cells (DCs) activate and license DCs via the CD40L–CD40 axis. Licensed DCs then present HLA class I-restricted neoantigens to CD8^+^ T cells, and the CD70–CD27 axis sustainably activates CD8^+^ T cells, inducing the expression of molecules associated with tumor homing and cytotoxicity while suppressing checkpoint molecule expression. When the same DC presents both HLA class II and class I neoantigens, it simultaneously activates helper CD4^+^ helper T cells and CD8^+^ cytotoxic T lymphocytes (CTLs), while ensuring efficient DC licensing by CD4^+^ helper T cells. TCR: T cell receptor. Treg: Regulatory T cell.

Collectively, these findings have prompted a conceptual shift in our understanding of CD4^+^ helper T cell biology. The classical model, in which CD4^+^ helper T cells were regarded primarily as sources of cytokines that nonspecifically augment CTL responses, has largely been superseded by a DC licensing-centered paradigm (Figure 1). In this framework, the principal contribution of CD4^+^ helper T cells is the conditioning of DCs, which subsequently orchestrates the magnitude, persistence, and functional quality of antitumor CD8^+^ T cell immunity [16,25].

## 5. Helper Neoantigen-Reactive CD4^+^ Helper T Cells and the Tumor Microenvironment

Helper neoantigen-reactive CD4^+^ helper T cells play a pivotal role in enhancing and sustaining antitumor immunity within the tumor microenvironment (TME). Huff et al. investigated the therapeutic efficacy of major histocompatibility complex (MHC) class II-restricted neoantigen vaccines in a murine cancer model. In the PancVAX2 model, vaccination with MHC class I-restricted neoantigens alone failed to elicit measurable antitumor activity unless combined with immune checkpoint inhibitors (ICIs). In contrast, co-administration of MHC class II- and class I-restricted neoantigen vaccines induced potent antitumor responses even in the absence of ICIs. This therapeutic benefit was accompanied by a reduction in regulatory T cells and an increase in immunostimulatory macrophages within the TME [26].

Similarly, Brightman et al. demonstrated that adoptive transfer of in vitro-expanded, neoantigen-reactive memory-like CD4^+^ helper T cells elicited CD8^+^ CTL-dependent antitumor responses, even in tumors lacking HLA class II expression in a murine tumor model. This effect was accompanied by increased intratumoral expression of IL-7 and IL-15, together with an expansion of PD-1^+^ CD8^+^ T cells within the TME [27].

Cui et al. identified tertiary lymphoid structures (TLS) in human lung cancer and demonstrated that the co-localization of follicular helper T cells and B cells within TLS was associated with improved patient survival. Their findings suggest a functional axis in which B cells present helper neoantigens to activate CD4^+^ helper T cells within human TLS. Furthermore, IL-21 produced by neoantigen-activated CD4^+^ helper T cells was shown to sustain CD8^+^ CTL function and promote antitumor immunity [28].

In addition, an ex vivo study using human samples by Lei et al. demonstrated that activated CD4^+^ helper T cells selectively engage classical type 1 dendritic cells (cDC1s), generating “helped cDC1s” that may facilitate their recruitment into tumor tissues. Single-cell analyses of human melanoma specimens further revealed that the presence of helped cDC1s was associated with enhanced priming and intratumoral accumulation of T helper 1-polarized CD4^+^ helper T cells and CD8^+^ CTLs, ultimately correlating with improved clinical outcomes [29].

Collectively, these findings indicate that the functions of CD4^+^ helper T cells extend well beyond preventing CD8^+^ CTL exhaustion and sustaining their proliferation and activation through dendritic cell licensing. As reviewed by Laidlaw et al., CD4^+^ helper T cells also reinforce CD8^+^ CTL effector function through IL-21 production and promote the generation of durable memory populations, including tissue-resident memory T cells [30].

The characteristics and functional significance of helper neoantigen-reactive CD4^+^ helper T cells in human tumors have been further elucidated by Veatch et al. Using single-cell transcriptomic analyses combined with neoantigen-reactive T-cell receptor clonotype profiling in melanoma, they identified CXCL13-expressing neoantigen-specific CD4^+^ helper T cells comprising at least two distinct subsets: follicular helper T cell-like cells and a cytotoxic-like population expressing TIM-3, cytolytic molecules, and IFN-γ. Notably, a higher frequency of CXCL13^+^ CD4^+^ helper T cells correlated with improved patient survival. These cells were also associated with CXCL9/10/11^+^ IL-15^+^ pro-inflammatory macrophages and CD8^+^ T cells and exhibited spatial co-localization with B cells within the TME, suggesting their central role in orchestrating coordinated antitumor immune responses [31].

Taken together, these findings indicate that neoantigen-reactive CD4^+^ helper T cells play a central role in shaping a coordinated antitumor immune network within the TME.

## 6. Antitumor Effects of Helper Neoantigen Vaccines

Despite the growing clinical application of neoantigen-based cancer vaccines, evidence supporting an optimal algorithm for selecting helper antigens to be combined with HLA class I-restricted neoantigen vaccines remains scarce. To augment vaccine-induced immune responses, nonspecific helper antigens, commonly referred to as universal helper immunogenic carrier proteins, have historically been co-administered with HLA class I-restricted peptide vaccines. These carrier proteins, exemplified by Keyhole Limpet Hemocyanin (KLH), facilitate the activation of CD4^+^ T helper cells and thereby enhance overall vaccine immunogenicity [32].

In a recent study, De Graaf et al. reported the results of a comparative analysis of a universal helper vaccine and a helper neoantigen vaccine in a murine tumor model, in which both vaccines were administered in combination with a neoantigen vaccine designed to activate CD8^+^ CTLs [33]. Specifically, they evaluated the antitumor activity of an MHC class I-restricted neoantigen vaccine administered either alone or in combination with a helper antigen capable of activating CD4^+^ helper T cells. The helper antigens consisted of either a neoantigen-derived helper epitope or a nonspecific helper antigen. Although both helper antigen vaccine strategies enhanced CD8^+^ T cell expansion, the neoantigen-specific helper vaccine induced significantly greater antitumor activity. Moreover, the helper neoantigen vaccine was superior in suppressing tumor growth and prolonging survival. Importantly, these effects were shown to be dependent on CD4^+^ helper T cells. These findings suggest that activation of neoantigen-specific CD4^+^ helper T cells may provide more effective support for the generation and maintenance of antigen-specific CD8^+^ CTL responses than activation of nonspecific CD4^+^ helper T cells.

From 2022 onwards, we have incorporated HLA class II-restricted helper neoantigen vaccines into our neoantigen vaccination strategy in combination with HLA class I-restricted neoantigen vaccines. We reported retrospective analyses evaluating the safety and scientific rationale of this approach. In a study published in 2023, we investigated the immunological effects of DC vaccines incorporating helper neoantigens in four patients with solid tumors [34]. In this study, HLA class II-binding long peptides containing HLA class I-restricted neoantigen epitopes (short peptides) were employed. This vaccination strategy successfully induced both neoantigen-reactive CD8^+^ T-cell and CD4^+^ T-cell responses, demonstrating the capacity of long peptides to activate cellular immunity through both HLA class I and class II pathways. Furthermore, long peptides containing multiple HLA class I-restricted neoantigens were shown to elicit immune responses against multiple CD8^+^ T-cell epitopes simultaneously. In a subsequent study reported in 2024, DC vaccines incorporating both helper neoantigens and HLA class I-restricted neoantigen peptides were administered to five patients with breast cancer in a postoperative setting with the aim of preventing disease recurrence [35]. A total of 17 helper neoantigen peptides were employed, and neoantigen-reactive lymphocyte responses were detected following vaccination for 10 of these peptides. By contrast, among 15 HLA class I-restricted neoantigen peptides tested, vaccine-induced immune responses were observed for only four peptides. These findings suggest that helper neoantigens possess superior immunogenicity and may play a pivotal role in the induction of robust antitumor immune responses. In addition, T-cell receptor repertoire analyses of peripheral blood mononuclear cells obtained after vaccination revealed the expansion of multiple T-cell clones carrying specific T-cell receptor sequences. These findings further support the immunogenic potential of neoantigen-based vaccination and provide evidence of vaccine-induced clonal T-cell expansion. Future clinical studies involving larger patient cohorts are warranted to further evaluate the clinical efficacy, immunogenicity, and therapeutic potential of helper neoantigen vaccines in cancer immunotherapy.

CD4^+^ helper T cells promote the activation and licensing of DCs, thereby preventing CD8^+^ CTL exhaustion and facilitating the development of long-term immune memory. Accordingly, helper neoantigen vaccines are expected to enhance the generation and maintenance of memory neoantigen-reactive CD8^+^ CTLs. Consistent with this concept, Hu et al. reported that personalized neoantigen vaccination incorporating helper neoantigens induced persistent neoantigen-specific T-cell responses in patients with melanoma, including CD8^+^ T cells exhibiting a memory-like phenotype [36].

Collectively, these findings indicate that the incorporation of HLA class II-restricted helper neoantigens into vaccine design enhances overall vaccine immunogenicity and promotes the generation of durable antitumor immune responses. In particular, long-peptide helper neoantigens can simultaneously stimulate neoantigen-reactive CD4^+^ and CD8^+^ T cells, thereby improving both the magnitude and quality of vaccine-induced immunity.

## 7. Strategies for Incorporating Helper Neoantigen Vaccines into Vaccine Design

**Figure 2:**
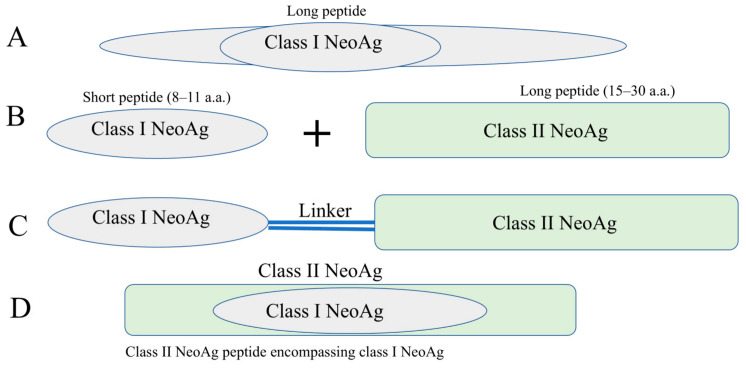
Design of HLA class I and class II neoantigen peptides for vaccine development. The figure shows four possible structural schemes for a vaccine capable of simultaneously activating CD8^+^ T cells and CD4^+^ T cells by incorporating HLA class I and class II neoantigens (NeoAg) into a vaccine. (A) Long peptide design containing HLA class I NeoAg sequences with the potential to incidentally generate HLA class II NeoAg. (B) Combination of HLA class I antigens and unrelated class II NeoAg. (C) HLA class I and class II NeoAg linked by a linker. (D) Incorporation of the HLA class I NeoAg sequence within class II NeoAg. a.a., amino acid.

Several strategies for combining an HLA class I-binding neoantigen peptide vaccine with an HLA class II neoantigen peptide vaccine are illustrated in Figure 2. In the context of DC-based vaccination, Method A involves the design of a long peptide containing an HLA class I-restricted neoantigen epitope, where the long peptide may also contain an HLA class II-binding neoantigen epitope. An example of this approach can be found in the vaccine design reported by Ott et al. [8]. Method B involves the separate design and co-administration of an HLA class I-restricted neoantigen short peptide and an HLA class II-restricted neoantigen long peptide. In this approach, the class I and class II neoantigens may be either related or unrelated. Method C involves linking an HLA class I-restricted neoantigen epitope to an HLA class II-restricted neoantigen epitope through a peptide linker. A key distinction among these approaches is that, in Method B, HLA class I- and class II-restricted neoantigens may be presented by different DCs, whereas in Methods C and D, the same DC can simultaneously present both neoantigens and activate neoantigen-reactive CD8^+^ and CD4^+^ T cells. Consequently, these approaches may promote more efficient DC licensing and elicit a more synergistic antitumor immune response.

Method A utilizes long peptides containing Class I neoantigens for vaccination, which may incidentally elicit CD4^+^ T-cell responses in addition to the intended CD8^+^ T-cell responses. Most neoantigen vaccines developed to date have adopted this strategy. However, because the MHC class II-binding affinity of the long peptides is not evaluated during vaccine design, this approach is straightforward but lacks precision in inducing helper T-cell responses. In contrast, Methods B, C, and D all combine MHC class I- and class II-restricted neoantigens, thereby providing rational strategies for coordinating CD8^+^ and CD4^+^ T-cell immunity. Method B represents the simplest combinatorial approach and has been employed in our previously reported clinical studies. Nevertheless, its success depends on ensuring that both neoantigens are presented by the same dendritic cell to achieve optimal CD4^+^ T-cell help for CD8^+^ T-cell priming.

Method C is a particularly promising strategy that has recently demonstrated robust antitumor efficacy in preclinical mouse models. As reported by Dolina et al. and Zhang R et al., Method C-type vaccines which consist of HLA class I-restricted and class II-restricted neoantigens linked by a linker tend to induce a stronger and broader immune response because the same antigen-presenting cells activate both neoantigen-reactive CD8^+^ T cells and CD4^+^ T cells [37,38]. Dolina et al. demonstrated that a neoantigen long-peptide vaccine containing linked MHC class I and class II epitopes can activate both neoantigen-reactive CD4^+^ and CD8^+^ T cells and induce significant tumor suppression in the SCC VII murine tumor model. By contrast, vaccines designed to activate only CD4^+^ or CD8^+^ T cells exhibited limited antitumor efficacy. Moreover, the linked CD4^+^/CD8^+^ neoantigen vaccine retained antitumor activity, even in ICI-resistant tumor models, highlighting the therapeutic potential of coordinated activation of both T-cell subsets [37]. Furthermore, Zhang et al. reported in murine tumor models that a neoantigen vaccine expressing- Class I- and Class II-restricted peptides linked by a peptide linker elicited greater CD8^+^ T-cell activation, epitope spreading and tumor infiltration, enhanced CD4^+^ T cell-dependent dendritic cell (DC) activation and tumor infiltration, and produced superior antitumor efficacy, compared with vaccination using either epitope alone [38]. This method is suitable for clinical application, and future clinical trials are anticipated.

**Figure 3:**
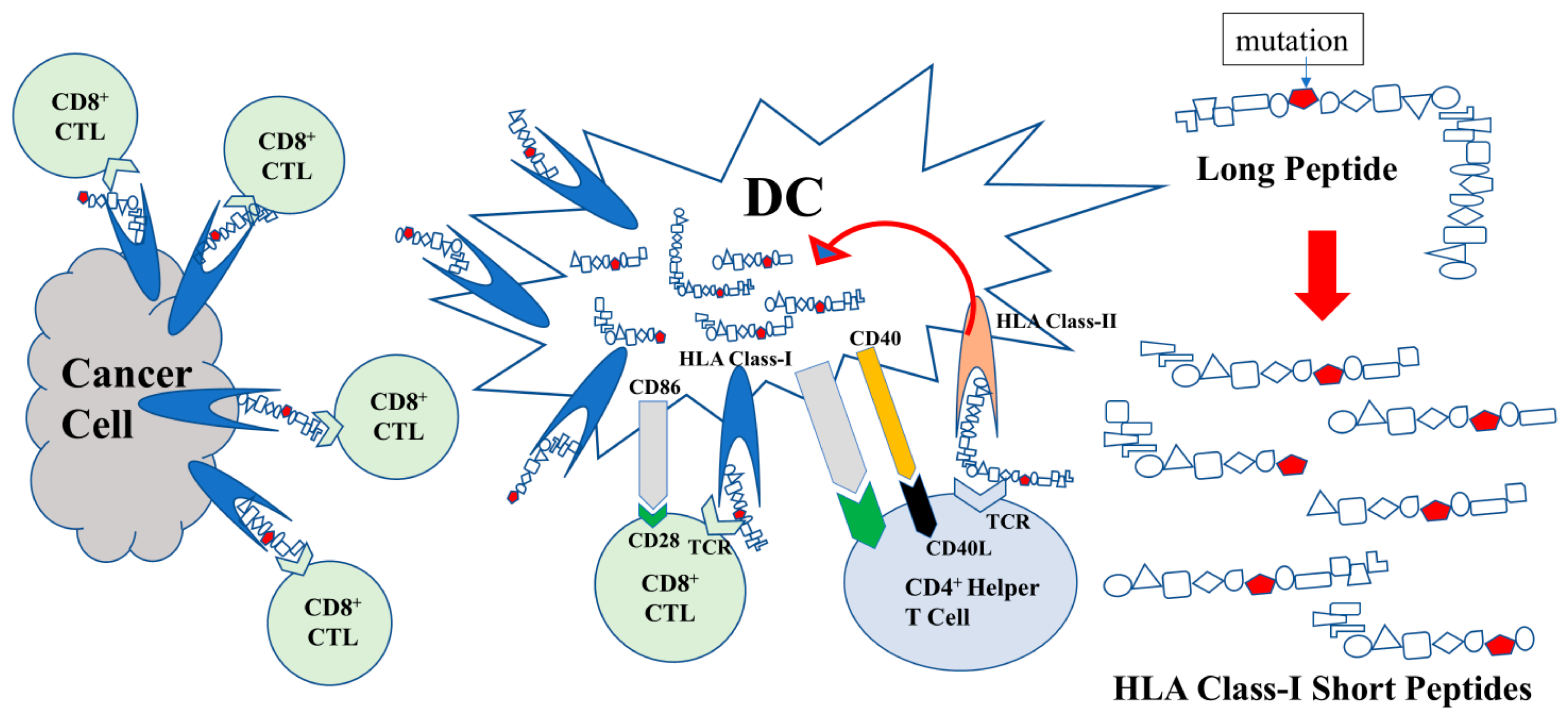
HLA class II long peptide encompassing HLA class I neoantigen epitope can stimulate multiple HLA class I neoepitope-reactive cytotoxic T lymphocytes (CTLs). HLA class II neoantigens containing multiple HLA class I neoantigen epitopes are taken up by dendritic cells (DCs), where they activate HLA class II neoantigen-reactive CD4^+^ T cells as well as multiple HLA class II neoantigen-specific CD8^+^ T cells. Representative examples of this mechanism are described in References [33,34]. TCR: T cell receptor.

Method D uses an HLA class II-restricted neoantigen long peptide that contains an embedded HLA class I-restricted neoantigen epitope. As reported in our previous studies, we have evaluated the immunological and clinical efficacy of neoantigen-pulsed DC therapy using monocyte-derived DCs loaded with HLA class II-binding neoantigen long peptides containing embedded HLA class I-restricted neoantigen epitopes, corresponding to Method D [34,35,39]. Implementation of this strategy requires the identification of both HLA class I-restricted neoantigen peptides and HLA class II-restricted neoantigen peptides (in our study, HLA-DR-restricted epitopes), as well as the identification of HLA class II-restricted neoantigens that encompass HLA class I-restricted neoantigen epitopes. Furthermore, as shown in Figure 3, this approach has demonstrated that DCs pulsed with class II neoantigens containing multiple class I neoepitopes can simultaneously activate multiple neoantigen-reactive T-cell populations [34,35]. Although Method D is an effective approach, it is based on our experience with a small number of cases. To expand the range of cases to which this method can be applied in the future, it will be necessary to develop methods for predicting Class I neo-epitopes and the highly immunogenic Class II neoantigens that contain those epitopes. Method D also represents an attractive strategy, although its applicability may be restricted to specific settings. Further studies are therefore required to define the contexts in which this approach can be most effectively applied.

## 8. Helper Neoantigen Dendritic Cell Vaccine Therapy

DC-based vaccine therapy incorporating tumor-associated antigens has been investigated for several decades; however, its clinical efficacy has generally been limited [22,23]. Nevertheless, DC vaccination, including approaches using monocyte-derived DCs, has recently regained attention because of its well-established immunological activity and its potential as a platform for personalized immunotherapy [40,41].

However, reports on neoantigen-pulsed dendritic cell (DC) vaccines remain limited [42]. In a pioneering study, Carreno et al. first demonstrated the induction of neoantigen-specific CD8 T-cell responses following vaccination with HLA class I-restricted neoantigen peptide-pulsed DCs in three patients with melanoma [43]. Subsequently, Ding et al. conducted a pilot clinical trial using DCs pulsed with both HLA class I- and HLA class II (HLA-DR)-binding neoantigen peptides in patients with lung cancer [44]. In this study, the class I and class II neoantigens were administered as separate peptides without physical linkage (Figure 2, Method B), and detailed analyses of helper CD4 T-cell responses were not performed.

To our knowledge, no previous study has reported a neoantigen DC vaccine in which HLA class II-restricted helper neoantigens are physically linked to HLA class I-restricted neoantigens (Figure 2, Method D). Within the regulatory framework for cell-based immunotherapy, we developed the HLA Class I/II Hybrid Neoantigen Peptide-Pulsed Dendritic Cell Vaccine, in which HLA class I-restricted neoantigen short peptides are linked to HLA class II (HLA-DR)-binding helper neoantigen long peptides and subsequently loaded onto DCs. We have previously reported retrospective analyses demonstrating the safety and potential clinical efficacy of this approach [34,35,39].

Regardless of the vaccine platform employed, future prospective clinical trials incorporating helper neoantigens, together with comprehensive immunomonitoring of vaccine-induced CD4 T-cell responses, will be essential for establishing the therapeutic value of helper neoantigen-based cancer vaccines.

For the prediction of HLA class II-restricted helper neoantigens, computational algorithms such as NetMHCII-2.2 and NetMHCIIpan-3.1 were employed, and peptides with predicted HLA-DR binding affinities of less than 50 nM were selected for vaccine preparation [34,35,45]. In these studies, no serious adverse events, including immune-related adverse events, were observed. Furthermore, both HLA class I-restricted and HLA class II-restricted neoantigen-specific immune responses were successfully induced, accompanied by measurable antitumor activity. Analysis of patients treated with DCs pulsed with helper neoantigen long peptides containing embedded HLA class I-restricted neoantigen epitopes further demonstrated the simultaneous activation of both HLA class I neoantigen-reactive CD8^+^ T cells and class II neoantigen-reactive CD4^+^ T cells, as reported in multiple studies [34,35,39].

As described in our previous reports, neoantigen-pulsed DCs are administered by ultrasound-guided intranodal injection [34,35]. Following intradermal or subcutaneous administration of DC vaccines, only a small proportion of the injected DCs migrate to regional lymph nodes, thereby limiting opportunities for interactions between neoantigen-presenting DCs and neoantigen-reactive lymphocytes. By contrast, our intranodal injection technique avoids major hilar vascular structures and causes minimal disruption of lymph node architecture. Because systemic lymphocytes continuously traffic through the medullary region of the lymph node, where DCs are injected, this approach is expected to increase the frequency of encounters between neoantigen-presenting DCs and neoantigen-reactive lymphocytes, thereby enhancing vaccine efficacy [46].

Helper neoantigen long peptides containing HLA class I-restricted neoantigen epitopes are internalized by DCs and incorporated into intracellular peptide pools. In addition to being processed and presented on HLA class II molecules, these peptides may serve as a sustained source of neoantigens for cross-presentation on HLA class I molecules. This strategy may therefore provide a more efficient means of neoantigen loading than conventional approaches relying solely on exogenously loaded HLA class I-binding short peptides. Furthermore, when the helper neoantigen long peptide contains embedded HLA class I-restricted neoantigen epitopes, DC licensing may facilitate both long-term activation of CTLs and activation of helper neoantigen-reactive CD4^+^ T cells, thereby promoting a coordinated and durable antitumor immune response.

## 9. Prediction and Rational Design of Helper Neoantigens: Emerging Challenges and Future Directions

Moving forward, several critical challenges must be addressed to fully realize the therapeutic potential of helper neoantigen vaccines. These include: (1) AI-assisted prediction of immunogenic helper neoantigens and rational vaccine design; (2) evaluation and mitigation of regulatory T cell (Treg) induction; (3) optimization of synergistic strategies with CD8^+^ T cell-targeting neoantigen vaccines; (4) advancement of immune monitoring technologies; (5) development of vaccine platforms capable of inducing B-cell responses and tertiary lymphoid structures (TLSs); (6) rational combination with immune checkpoint inhibitors [47,48]; and (7) loss or downregulation of MHC class I expression on tumor cells.

Among these challenges, the identification of HLA class II-restricted helper neoantigens remains particularly difficult. The limited accuracy of current HLA class II binding prediction algorithms, the discordance between predicted HLA binding affinity and actual immunogenicity, variability in antigen processing and presentation, intratumoral heterogeneity, and extensive HLA polymorphism all contribute to the complexity of helper neoantigen prediction. To overcome these limitations, several next-generation approaches are rapidly emerging, including AI-driven neoantigen prediction, multimodal immunopeptidomics, single-cell spatial transcriptomics, and T-cell receptor (TCR) repertoire-guided vaccine design. Recently, Huber et al. developed NeoDisk, an integrated neoantigen prediction platform that combines genomic, transcriptomic, and immunopeptidomic data. Compared with conventional prediction pipelines, NeoDisk demonstrated improved identification of highly immunogenic neoantigens, highlighting the potential of multimodal approaches for next-generation personalized vaccine design [49].

Another important issue is the potential induction of immunosuppressive CD4^+^ T-cell subsets following helper neoantigen vaccination. Although increases in Tregs have been widely reported as a physiological negative-feedback mechanism after cancer vaccination [50,51], such findings have not yet been documented in clinical studies of neoantigen vaccines. Nevertheless, robust vaccine-induced immune activation could theoretically promote compensatory Treg expansion. If clinically significant Treg induction is observed, combining vaccination with low-dose cyclophosphamide—which selectively reduces Tregs while maintaining an excellent safety profile—may represent a practical strategy to enhance therapeutic efficacy and is currently recommended in several vaccination protocols [52].

In addition to Tregs, vaccine dose itself may critically influence the quality of CD4^+^ T-cell responses. Using a murine tumor model, Sultan et al. recently demonstrated that a low-dose helper neoantigen vaccine elicited effective antitumor immunity, whereas a high-dose vaccine preferentially induced cytotoxic CD4^+^ T cells, which paradoxically impaired antitumor immune responses. These findings suggest that quantitative aspects of antigen exposure may shape CD4^+^ T-cell differentiation and underscore the importance of defining the optimal dosing regimen for helper neoantigen vaccines. Further mechanistic and clinical studies are therefore warranted to determine vaccine doses that maximize durable antitumor immunity while minimizing potentially suppressive immune responses [53].

Furthermore, the loss or downregulation of HLA class I expression, which is critical for CD8^+^ T-cell-mediated recognition of neoantigens, represents an important immune evasion mechanism that cannot be overlooked. Loss of MHC class I expression has been reported in a wide variety of human cancers to varying degrees, potentially impairing antitumor immunity by reducing the presentation of neoantigens on the surface of cancer cells [54]. In addition to direct loss-of-function mutations affecting HLA class I molecules, several mechanisms have been identified that indirectly impair MHC class I expression and function. These mechanisms include not only genetic alterations but also epigenetic changes, such as DNA methylation and histone modifications, which regulate the synthesis of MHC class I molecules and their processing and transport to the cell surface. Several exploratory clinical studies have investigated therapeutic strategies targeting these mechanisms, and although limited in number, some have demonstrated promising potential for restoring HLA class I expression [55]. However, evidence from large-scale clinical trials is still lacking. Further research is therefore warranted to establish effective approaches for overcoming reduced HLA class I expression, particularly to enhance the efficacy of neoantigen-based cancer vaccines.

**Figure 4:**
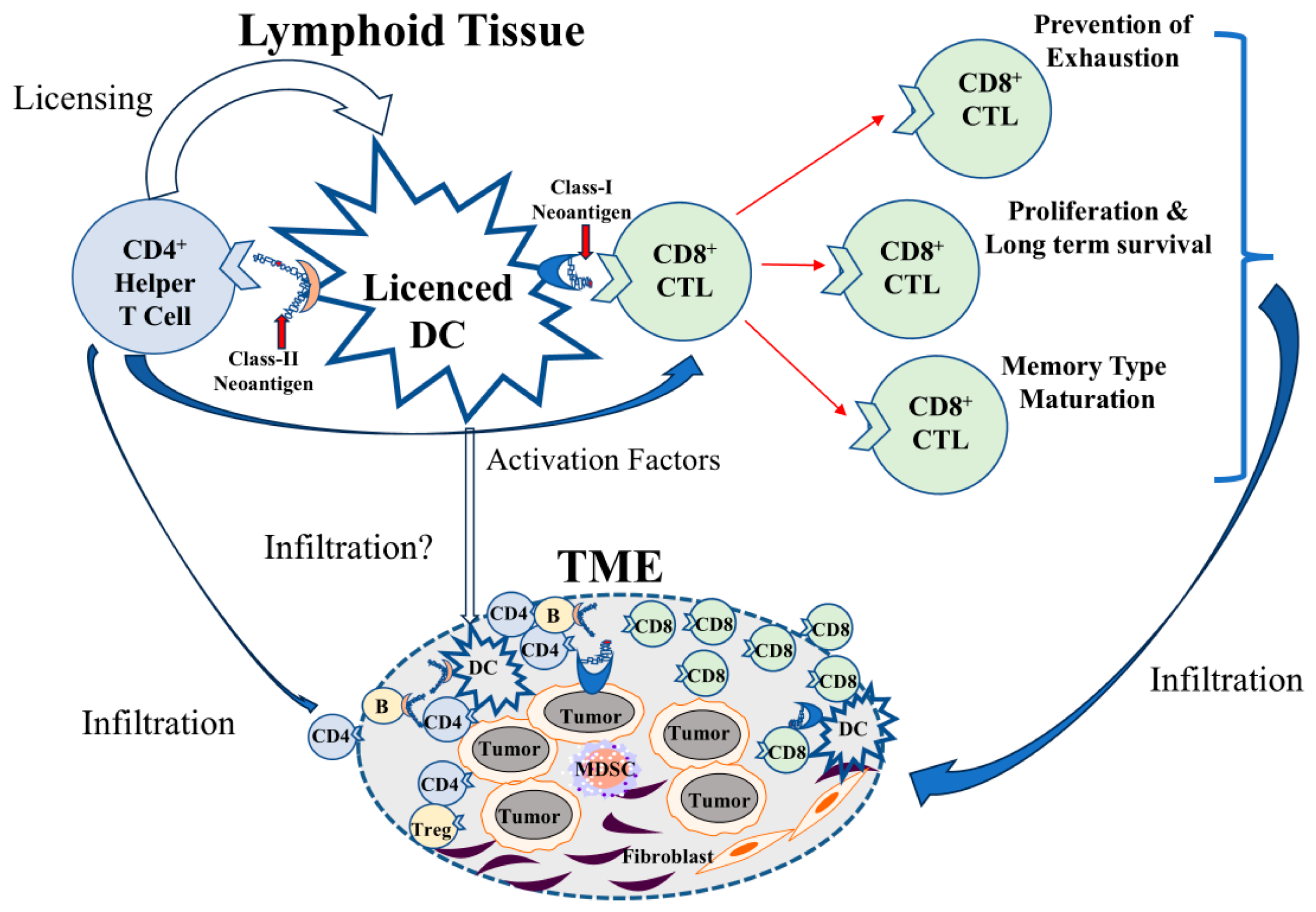
The central role of neoantigen-specific CD4^+^ helper T cells in antitumor immunity. Dendritic cells (DCs) licensed by CD4^+^ helper T cells not only present antigens to CD4^+^ and CD8^+^ T cells, but also prevent CD8^+^ cytotoxic T cells (CTLs)’ exhaustion, promote their proliferation, extend their lifespan, and support their differentiation into memory T cells. Furthermore, they enhance the accumulation of antitumor CD8^+^ CTLs and DCs within the tumor microenvironment (TME), thus playing a central role in antitumor immunity. B: B cell; MDSC: myeloid-derived suppressor cell.

In parallel with advances in prediction algorithms, vaccine development is evolving from simple neoepitope prediction toward the rational design of helper neoantigen vaccines based on immunological principles. In particular, long peptides containing both HLA class I- and class II-restricted neoepitopes have been employed to promote coordinated activation of CD8^+^ CTLs and CD4^+^ helper T cells. In addition, a “helper-first” vaccine design strategy based on highly immunogenic HLA class II neoepitopes has been proposed as a means of inducing more durable antitumor immune responses [17,56,57]. In this context, CD4^+^ helper T cells should no longer be regarded merely as auxiliary cells that support CD8^+^ T-cell responses, but rather as central regulators of antitumor immunity through their roles in DC activation, maintenance of CD8^+^ T-cell function, and promotion of epitope spreading. This concept should be incorporated into the design of future immunotherapeutic strategies (Figure 4).

Further studies are needed to improve methods for helper neoantigen prediction and to establish rational approaches for helper neoantigen vaccine development. In addition, large-scale clinical trials evaluating immunotherapies targeting both helper neoantigens and HLA class I-restricted neoantigens are warranted to determine their clinical utility and therapeutic potential.

## 10. Conclusions

Although many challenges remain in establishing Helper Neoantigen Vaccine therapy with effective and sustained antitumor effects, it is also necessary to further elucidate the role of Helper Neoantigen-reactive T cells—which can direct a diverse array of immune cells—in antitumor immune responses.

## Data Availability

No new data were created or analyzed in this study.

## Abbreviations

CTL	Cytotoxic T Lymphocyte
DC	Dendritic Cell
ICI	Immune Checkpoint Inhibitor
IFN	Interferon
IL	Interleukin
TME	Tumor Microenvironment
TLS	Tertiary Lymphoid Structure
HLA	Human Leukocyte Antigen
MHC	Major Histocompatibility Complex
